# Bacterial Quorum-Quenching Lactonase Hydrolyzes Fungal Mycotoxin and Reduces Pathogenicity of *Penicillium expansum*—Suggesting a Mechanism of Bacterial Antagonism

**DOI:** 10.3390/jof7100826

**Published:** 2021-10-02

**Authors:** Shlomit Dor, Dov Prusky, Livnat Afriat-Jurnou

**Affiliations:** 1 Migal-Galilee Research Institute, Kiryat Shmona 11016, Israel; shlomitd@migal.org.il; 2 Department of Postharvest Science, Agricultural Research Organization, Rishon LeZion 7505101, Israel; 3 Faculty of Sciences and Technology, Tel-Hai Academic College, Upper Galilee 1220800, Israel

**Keywords:** *Penicillium expansum*, patulin, mycotoxin, quorum-quenching (QQ) lactonase, fungal pathogens

## Abstract

*Penicillium expansum* is a necrotrophic wound fungal pathogen that secrets virulence factors to kill host cells including cell wall degrading enzymes (CWDEs), proteases, and mycotoxins such as patulin. During the interaction between *P. expansum* and its fruit host, these virulence factors are strictly modulated by intrinsic regulators and extrinsic environmental factors. In recent years, there has been a rapid increase in research on the molecular mechanisms of pathogenicity in *P. expansum*; however, less is known regarding the bacteria–fungal communication in the fruit environment that may affect pathogenicity. Many bacterial species use quorum-sensing (QS), a population density-dependent regulatory mechanism, to modulate the secretion of quorum-sensing signaling molecules (QSMs) as a method to control pathogenicity. N-acyl homoserine lactones (AHLs) are Gram-negative QSMs. Therefore, QS is considered an antivirulence target, and enzymes degrading these QSMs, named quorum-quenching enzymes, have potential antimicrobial properties. Here, we demonstrate that a bacterial AHL lactonase can also efficiently degrade a fungal mycotoxin. The mycotoxin is a lactone, patulin secreted by fungi such as *P. expansum*. The bacterial lactonase hydrolyzed patulin at high catalytic efficiency, with a *k_cat_* value of 0.724 ± 0.077 s^−1^ and K_M_ value of 116 ± 33.98 μM. The calculated specific activity (*k_cat_*/K_M_) showed a value of 6.21 × 10^3^ s^−1^M^−1^. While the incubation of *P. expansum* spores with the purified lactonase did not inhibit spore germination, it inhibited colonization by the pathogen in apples. Furthermore, adding the purified enzyme to *P. expansum* culture before infecting apples resulted in reduced expression of genes involved in patulin biosynthesis and fungal cell wall biosynthesis. Some AHL-secreting bacteria also express AHL lactonase. Here, phylogenetic and structural analysis was used to identify putative lactonase in *P. expansum*. Furthermore, following recombinant expression and purification of the newly identified fungal enzyme, its activity with patulin was verified. These results indicate a possible role for patulin and lactonases in inter-kingdom communication between fungi and bacteria involved in fungal colonization and antagonism and suggest that QQ lactonases can be used as potential antifungal post-harvest treatment.

## 1. Introduction

Microorganisms associated with the fruit microbiome are found on the surfaces (epiphytes) or in the tissues of the fruit (endophytes). The recent knowledge gained from microbial community analysis indicates location dependence and is relevant to biological control to prevent post-harvest fruit pathology [1]. The demand to study the epiphytic microbiome is increasing in light of the understanding that raw-eaten plants seem to be a source for microbes that are a part of the gut microbiome and a source for pathogens that might play a role in human health [2]. Among other microbes, filamentous fungi are found in raw food, and most of them produce metabolites that are of risk to human health [3,4,5]. Some of them are also associated with human infections [3]. For example, the plant’s pathogenic species *P. citrinum*, *P. chrysogenum*, *P. digitatum*, *P. marneffei*, and *P. expansum* can cause human infection through inhalation and sometimes ingestion [4], causing necrotizing esophagitis, endophthalmitis, keratitis, and asthma [3,4].

*P. expansum* is a necrotrophic wound fungal pathogen that secrets various virulence factors to kill host cells, including cell wall degrading enzymes (CWDEs), proteases, and also produces mycotoxins such as patulin [5]. During the interaction between *Penicillium expansum* and its fruit host, these virulence factors are strictly modulated by intrinsic regulators and extrinsic environmental factors [5,6,7]. *P. expansum* also has a cytotoxic effect that can lead to health risks in agriculture workers [8]. In recent years, there has been a rapid increase in the research towards understanding the molecular mechanisms, including the involvement of mycotoxins in pathogenicity of *P. expansum*, especially after sequencing of the genomes of *P. expansum* and closely related *Penicillium* species [5]. Patulin is a lactone-based mycotoxin produced by *P. expansum*, most commonly found in colonized apples. The amount of patulin in apple products is generally viewed as a measure of apple quality. It reacts with thiol groups of macromolecules and causes serious health problems in humans and animals [8,9]. Due to the high toxicity of patulin, many toxicological regulatory organizations worldwide have set a maximum limit for patulin levels in foods, and studying the genes and enzymes involved in its biological degradation are of great interest [9,10]. Furthermore, it was indicated that patulin is involved in fungal pathogenicity [5,6,7].

Quorum sensing (QS) is one of the most studied regulatory mechanisms that enable bacteria to monitor their population density, integrate intercellular signals, and coordinate gene expression to benefit the bacterial community in various environments [11,12]. QS plays a key role in the host microbiome as it enables bacteria to synchronize their behavior upon changes in population composition [13]. By sensing the extracellular concentration of secreted auto-inducer molecules, quorum-sensing signaling molecules (QSMs), the expression of various genes, such as genes involved in biofilm formation, antibiotics production, and virulence factors are affected [14]. QS systems show a wide range of signaling diversity, and different bacteria secrete different signaling molecules sensed by cognate receptors [15]. For example, N-acyl homoserine lactones (AHLs) are the most common auto-inducers of Gram-negative bacteria [11,16]. Many bacterial pathogens utilize AHLs to coordinate pathogenicity [17], including *Pantoea stewartii*, *Erwinia carotovora*, *Pseudomonas syringae*, and *Xanthomonas campestris* [18]. QS systems are appealing antimicrobial therapeutic targets, mainly since they regulate virulence gene expression in bacterial pathogens [19,20,21]. Targeting QS will attenuate the production of virulence factors without exerting selective pressure and potentially lower the chances of resistance development19-21]. Strategies that target QS are named quorum-quenching (QQ) strategies [19,20,21]. Interestingly, patulin can act as a quorum-sensing (QS) inhibitor molecule; for example, in *Pseudomonas aeruginosa* it downregulated QS-regulated genes [22]. Patulin also inhibited QS-regulated biofilm formation in *Methylobacterium oryzae* (a Gram-negative bacteria) and affected bacterial cell numbers [23]. Co-growth of *Methylobacterium oryzae* and *P. expansum* spores induced a differential gene expression of genes involved in patulin biosynthetic pathway clusters (such as the gene coding for isoepoxydon dehydrogenase) [24]. Therefore, patulin production may play a role in inter-kingdom communication.

Several enzymes which degrade bacterial AHLs were characterized, such as acylases [25] and lactonases [26,27]. AHL lactonases were identified in both Gram-negative and Gram-positive bacteria [28,29,30], and in mammalians [31]. AHL lactonases proficiently hydrolyze the lactone ring in AHLs, leading to inhibition of QS related functions such as biofilm, virulence factors, and infections [32,33,34,35,36,37,38,39]. Since these lactonases are widely conserved in various bacterial species, and have a variable substrate range [30], several hypotheses were proposed regarding their biological role. Some suggest that AHL-lactonases self-regulate QS signaling within the same species, supported by the evidence that AHL-producing bacterial strains can also degrade them [38]. However, as bacteria that do not have AHL-based QS also express AHL lactonases, a selective advantage in attenuate QS of neighboring bacteria is another possibility [39]. Filamentous fungi also possess AHL lactonase activity. Intracellular AHL lactonases were identified in *Coprinopsis* *cinerea* and characterized, these fungal lactonases belong to the metallo-β-lactamase family (MBL, PF00753) exhibited similar AHL hydrolyzing activity as AiiA from *Bacillus thuringiensis* [40]. Another example is that forest root-associated fungi are able to degrade AHLs [41]. 

Fungi and bacteria co-exist in various habitats, and are thought to be engaged in inter-kingdom communications such as QSM by cross detection or degradation [42]. Fungi secrete various QS molecules, such as farnesol in the pathogenic fungus *Candida albicans* [43,44], and alcohols derived from aromatic amino acids [45]. QS molecules that play a role in fungal pathogenicity were studied in yeasts and filamentous fungi, such as *Candida albicans*, *Candida dubliniensis*, *Aspergillus niger*, *Aspergillus nidulans*, and *Fusarium graminearum* [45]. Interestingly, *Fusarium* mycotoxins can interact and inhibit bacterial QS communication [46]. 

The apple microbiome depends on many factors such as genotype, management practices, and more [1,47,48,49,50,51]. A recent study indicated that the abundance and distribution of bacterial phyla in the “Royal Gala” apple fruit were consistent in most examined countries [1]. The most abundant bacterial genera were *Sphingomonas*, *Erwinia*, *Pseudomonas*, *Bacillus*, unidentified *Oxalobacteraceae*, *Methylobacterium*, and unidentified *Microbacteriaceae* [1]. In terms of the apple fungi community, in all countries, the most dominant phyla were *Ascomycota* (79.8%) then *Basidiomycota* (9.3%) [1]. Two of the major microbial pathogens that affect apple production are the fungi *Penicillium* *expansum*, causing the post-harvest disease blue mold [5] from the *Ascomycota* phyla, and the Gram-negative bacterial phytopathogen *Erwinia amylovora* from *Erwinia*, the cause of fire-blight disease [52]. However, a deep understanding of the molecular mechanisms involving the epiphytic microbial population’s interaction is still needed [1]. 

Here, the activity of a purified bacterial lactonase with the fungal mycotoxin patulin was detected. Furthermore, the enzyme presented an inhibitory effect on *P. expansum* cultures when applied before apple infection, including downregulation of genes expression. To maintain enzymes’ stability upon its addition to fungal cultures and during infection, we used a stabilized mutant of PPH (Parathion Protein Hydrolase) [26] from *Mycobacterium tuberculosis*. PPH-G55V presented improved residual activity at high temperatures [53]. Therefore it is more suitable for biotechnological applications, testing lactonases activity, and their effects *in cultures*. Moreover, the identification and characterization of a new lactonase, active with patulin, from *P. expansum* is presented. The results indicate a possible role for patulin and its degradation by lactonases in inter-kingdom communication between fungi and bacteria. They further suggest QQ lactonases as potential antifungal post-harvest treatment as a strategy to lower fungal mycotoxins food contamination.

## 2. Materials and Methods

### 2.1. Fungal Growth Conditions

The plant pathogen *Penicillium expansum* Pe-21 isolated from decayed local cvs. Grand Alexander and Golden Delicious apples, provided by the Department of Postharvest Science of Fresh Produce, ARO, Volcani Center, Israel [54]. Isolate Pe-21 was used to study the activation of glucose oxidase and secretion of gluconic acid by *P. expansum* pathogenicity in apples [7,54]. Moreover, Pe-21 knockout established a connection between LaeA, a global regulator and the regulation of several secondary metabolite genes, including the patulin gene cluster [55]. Cultures were grown on potato dextrose agar (PDA) plates (Difco, Detroit, MI, USA) at room temperatures at the range of 22–24 °C in the dark. Mycelial growth and fruit inoculation were assays from one-week-old conidia, harvested from potato dextrose agar PDA plates. Conidia were harvested from PDA plates after adding 5 mL of sterile distilled water with 0.01% (*v*/*v*) Tween 20 (Sigma-Aldrich, Copenhagen, Denmark), gently rubbing the fungal spores, pulling the liquid together, and collected to 1.5 mL tubes. PDB (Potato Dextrose Broth) medium (Difco, Detroit, MI, USA) was used for growing liquid cultures. *P. expansum* spores are ellipsoidal, 3.0–3.5 μm long, and smooth-walled.

### 2.2. Recombinant Expression and Purification of Lactonases

We used a variant of a highly efficient AHL lactonase, PPH, from *M. tuberculosis* belonging to the PTE-like lactonase family[26]. We previously obtained PPH-G55V by using directed enzyme evolution [53]. The variant harbors one point mutation (Gly to Val at position 55) exhibited increased thermal stability and shelf life [53], essential criteria for biotechnological applications. pMAL-c4X-PPH-G55V vector was used for lactonase expression as a fusion protein with maltose binding protein (MBP). Its recombinant expression and purification were performed as previously described [53,56]. Briefly, freshly transformed *E.coli*-BL21 (DE3) cells with pMAL-c4xPPH-G55V, were inoculated in to 10 mL LB medium with 100 µg/mL ampicillin and 0.5 mM MnCl_2_. Cultures were grown at 37 °C, 170 rpm. Following overnight growth, cultures were added to 1 L LB medium for several hours at 30 °C, 170 rpm. When the cultures OD_60_ reached 0.6–0.8, expression was executed by the addition of 0.4 mM IPTG (Isopropyl β-d-1-thiogalactopyranoside) for overnight expression at 20 °C. Cells were harvested by centrifugation, and then suspended in lysis buffer containing 100 mM Tris-HCl pH 8.0, 100 mM NaCl, 100 µM MnCl_2_ and protease inhibitor cocktail (Sigma-Aldrich, Israel) diluted 1:500. Cultures were centrifuged and supernatants were passed through an amylose column (NEB, New England Biolabs, Ipswich, MA, USA) previously equilibrated with activity buffer (100 mM Tris pH 8.0, 100 mM NaCl, and 100 µM MnCl_2_). Protein was eluted with column buffer supplemented with 10 mM maltose, and loaded on a size exclusion chromatography (SEC) column, HiLoad^®^ 16/600 Superdex^®^ 75 pg column (GE Healthcare, Chicago, IL, USA), adapted for the AKTA fast protein liquid chromatography (FPLC) system and equilibrated with filtered column buffer. The purity of the fusion enzymes was established by 12% SDS-PAGE, and samples were stored at 4 °C.

The codon optimized sequence of putative lactonase from *P. expansum* (named PELa) was ordered from GenScript (New Jersey, NJ, USA) cloned into an expression vector, pMAL-c4X, at its *Eco*RI and *Pst*I sites. pMAL-c4X—PELa vector was used for lactonase expression as a fusion protein with maltose binding protein (MBP). Recombinant expression was performed in *E. coli*-BL21 (DE3) cells containing pGro7 plasmid (TAKARA, Shiga, Japan), for co-expression with GroEL/ES as a chaperon, as described in [26]. For this, pMal-PELa plasmid was transformed into *E. coli*-BL21 (DE3) cells, containing the pGro7 plasmid and plated on LB agar with 100 μg/mL ampicillin and 34 μg/mL chloramphenicol. These overnight cultures were used to inoculate (at 1:100 dilution) a fresh LB with 100 μg/mL ampicillin, 34 μg/mL chloramphenicol and 100 μM ZnCl_2_, and 0.05% (*w*/*v*) arabinose, to induce GroEL/ES expression. Cells were grown at 30 °C with shaking to reach an OD_600_ = 0.6–0.8, then final concentration of 0.4 mM IPTG was added to induce overexpression. Protein purification was performed as described for PPH-G55V.

### 2.3. Enzyme Kinetics Analysis

The activity of PPH–G55V (0.3 μM) with patulin was analyzed using UV detection of patulin [57]. For this 0.1 mM patulin in activity buffer: 100 mM Tris pH 7.5, 100 mM NaCl, 100 μM MnCl_2_ was used for an absorbance scan, from 240–310 nm, at 24 °C (BioTeK (Winooski, VT, USA), optical length of ~0.5 cm). The absorbance of patulin in activity buffer was measured at 278 nm in UV 96-well plates, with and without purified enzymes. Activity was monitored in a microtiter plate reader. Patulin’s (in activity buffer) extension coefficient was calculated from the preformed calibration curve using patulin in increasing concentrations (0–0.4 mM). PPH-G55V activity was tested with different patulin concentrations (ranging from 0 to 0.3 mM). Reactions were performed at the same concentration of organic solvent, regardless of substrate concentration. V_0_-enzyme initial rates were corrected for the background rate of patulin spontaneous hydrolysis in the absence of the enzyme. Kinetic parameters were obtained by GraphPad software as fitting initial rates directly to the Michaelis–Menten equation V_0_ = k_cat_[E]_0_[S]_0_/([S]_0_ + K_M_) [26]. Error ranges relate to the standard deviation of the data obtained from at least two independent measurements.

### 2.4. Addition of Purified Lactonase to P. expansum Liquid Culture

Purified lactonase at a final concentration of 2 μM was added to a 3 mL *P. expansum* culture in potato dextrose broth (PDB) medium containing 2.5 × 10^3^ spores, grown at 25 °C, 150 rpm. After 3 days, mycelium growth was visually evaluated, and mycelium fraction was weighted following centrifugation for 10 min at 10,000 rpm (fresh weight). Mycelia treated with the enzyme’s activity buffer was used as a control. Each treatment was consisted of 3 biological repeats.

### 2.5. Effect of Purified Lactonase on Spores Germination and Colony Growth

*P. expansum* conidia were harvested from 5-day-old PDA plates. Conidia harvesting was performed by spreading 5 mL of 0.01% (*v*/*v*) Tween 20 (Sigma-Aldrich, Copenhagen, Denmark) in sterile ddH_2_O (sterile double distilled water). Purified enzyme (2 μM) was added to 1 mL sterile water containing 2.5 × 10^3^ spores and incubated with shaking at 300 rpm at 25 °C. Spore germination was observed microscopically (Micros Lotus MCX51,Gewerbezone, Austria) at a magnification of 40×; every 60 min, 20 µL from the solution was examined with a hemocytometer (Assistant, Germany).

To test colony growth, *P. expansum* spores were collected from a colony that grew for 5 days. Spores (2 µL from 10^6^/mL solution in 1 mL of PDB medium) were incubated with shaking at 300 rpm in the presence of 2 μM PPH-G55V for 30 min at 25 °C. After incubation, a 10 µL aliquot of treated spores was spread on PDA plates and placed in the dark at room temperature for 48 h to test colony regeneration and development.

### 2.6. Pathogenicity Assay of P. expansum in Apples

*P. expansum* spores (10 μL of 2.5 × 10^3^) were incubated at 25 °C during 30 min with 2 μM purified enzyme in 1 mL final volume (with sterile ddH_2_O) previously to inoculation in fruits. Freshly harvested apples cv. “Golden Delicious” were surface-cleaned with 70% ethanol and wound-inoculated by puncturing 2 mm deep with a sterile needle. Conidial suspension (10 μL) and the enzyme were placed on the wound, and incubated at 24 °C. Disease colonization was monitored daily for disease symptoms and lesion diameter. In each treatment, five different apples were inoculated 3 times per apple at the equatorial axis (5 × 3 = 15 replications). Spores incubated in enzyme buffer (100 mM Tris-HCl pH 8.0, 100 mM M NaCl, and 100 µM MnCl_2_) without the enzyme were used as a control. To assess the effect of enzyme application time on disease development, apples were also sprayed with the purified lactonase, 30 min prior or post inoculation with spore suspensions on apples surface, and enzyme buffer was used as a control.

### 2.7. RNA Isolation and Quantitative Real-Time PCR (qPCR)

RNA was extracted from grinded mycelia or from the leading edge of the decayed infected apple tissue, as previously described [58]. RNA extraction was performed with a fungal total RNA purification kit (Norgen, Canada) according to the manufacturer’s protocol. cDNA was then synthesized. Using the Verso cDNA Synthesis Kit (ThermoFisher Scientific, Waltham, MA, USA). qPCR was performed using the LightCycler Instrument II (Roche, Basel, Switzerland) in 384-well plates. PCR amplification was performed with 1 ng/μL cDNA template in 4 µL of a reaction mixture (LightCycler 480 SYBR Green I Master, Roche) containing 250 nM primers final concentration. 

For qPCR analysis, the amplification program included one cycle of pre-incubation at 95 °C for 5 min, followed by 45 cycles of 95 °C for 10 s, 60 °C for 20 s, and 72 °C for 20 s followed by a melting curve analysis cycle of 95 °C for 5 s and 65 °C for 1 min. Relative quantification of all samples was normalized to 28 s expression levels and calculated using the ΔCt model [59]. The ΔCT value was determined by subtracting the CT results for the target gene from those for the endogenous 28 s control gene. As described by ΔCt target = Ct (reference gene) − Ct (target gene). Primer efficiency was established using serial dilutions of pooled cDNA and found to be equal to 1.98 (amplification factor per cycle). Efficiency was presumed to be the same for all samples. Therefore, the calculated expression ratio was: ratio = 1.98^ΔCt^. Each experiment was performed in three different biological replicates. For each biological sample the qPCR ran was conducted in four technical repetitions.

### 2.8. Sequence Identification, Alignment of Putative Lactonase, and Structure Modeling

Homologs were identified using the sequence of AiiA (WP_000216581.1), an AHL lactonase from *B. thuringiensis* previously characterized[30]. The search was performed with the protein-alignment BLAST (blastp) function in the NCBI nonredundant protein sequence database (nr), and included several available genomes of bacterial spices such as *Bacillus megaterium* (basionym: *Priestia megaterium)* and fungal species such as *P. expansum*, *Aspergillus clavatus, Penicillium digitatum, Pseudogymnoascus verrucosus*, and *Fonsecaea pedrosoi*. The sequences of identified putative homologs and previously characterized MBL (metallo-β-lactamase) superfamily AHL lactonase (ranging from 59% to 78% identity between homologs), were aligned in MEGA X software [60]. An alignment picture was created with the freely available software Jalview [61].

A sequence with 29.25% identity, 77% coverage, and E-value of 2 ×10^−21^ was found in the genome of *P. expansum (*XP_016600436.1) annotated as hypothetical protein PEX2_072460. The putative enzyme was named here PELa, for *P. expansum* Lactonase. To further validate that the newly identified fungal enzyme is a lactonase, structural comparison with solved structure of bacterial AHL lactonase was performed. For this, a 3D structural model was generated by submitting XP_016600436.1 amino acid sequence to an online sever SWISS-MODEL (https://swissmodel.expasy.org/, accessed on 29 August 2020). SWISS-MODEL is an automated software that calculates structural models based of known solved structures used as templates, and sequence-structure alignments [62]. Specifically, the solved structure of the AHL lactonase from *Alicyclobacillus acidoterrestris*, PDB (Protein Data Bank) number 6cgy was found as best hit by the server for modeling, and therefore it was used as a template for structural modeling of PELa. Next, the resulting structural model of PELa from *P. expansum* was aligned with the structure of AHL lactonase from *Alicyclobacillus acidoterrestris* (pdb 36cgy), using PyMOL Molecular Graphics System, Version 1.2r3pre, Schrödinger, LLC (New York, NY, USA).

### 2.9. Putative Lactonase from P. expansum Characterization

The codon-optimized sequence of putative lactonase from *P. expansum* (named PELa) was ordered synthetically from GenScript (New Jersey, USA) cloned into an expression vector, pMAL-c4X at its *Eco*RI and *Pst*I sites. The pMAL-c4X vector was used for expression as a fusion protein with maltose binding protein (MBP), and protein was expressed and purified as described above. Purified PELa (0.6 µM) was incubated with 0.3 mM patulin at various temperatures to determine the optimal temperature for hydrolytic activity. Samples collected at time 0 and after 2 min, were spectrophotometrically analyzed at 278 nm. The control sample (activity buffer; 100 mM Tris-HCl pH 7.5, 100 mM M NaCl, and 100 µM ZnCl_2_) was incubated under the same conditions, and values were subtracted from each corresponding test sample containing the enzyme. Readings of pre-incubation samples were subtracted from the reading of post-incubation samples. A total of 100% activity was defined as the activity at 25 °C. Each treatment was tested in triplicates. To test the optimal pH for activity, 0.6 µM of purified PELa was diluted in activity buffer adjusted to pH values ranging between 3.5 and 11 (100 mM acetate buffer for pH 4.5–5.5, phosphate buffer for pH 5.5–8.0, Tris buffer for pH 8.0–9.0). Enzyme activity was measured at 25 °C for 15 min (at higher temperatures, high spontaneous hydrolysis was observed), by adding 0.3 mM patulin, in the same buffer for each pH value. The spontaneous hydrolysis of patulin in enzyme-free activity buffer at each pH was subtracted from the hydrolysis measured in each corresponding test sample.

## 3. Results

### 3.1. Bacterial QQ Lactonase Degrades Patulin, Inhibits Apples Infection, and Modulates Gene Expression in P. expansum during Infection

**Figure 1:**
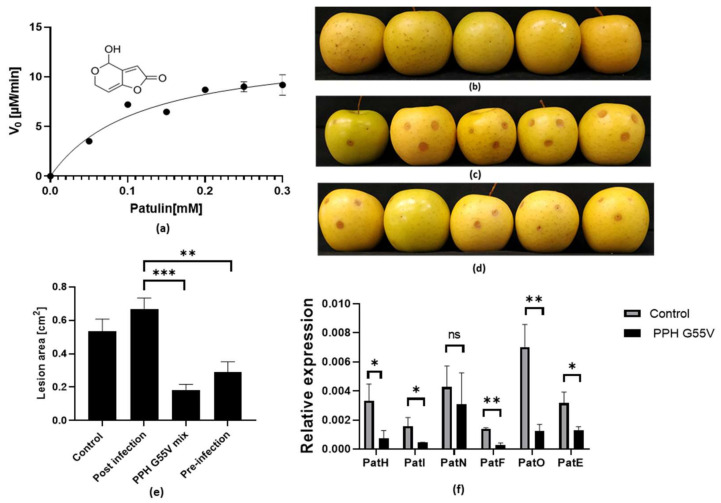
Bacterial lactonase degrades patulin in vitro, inhibits apples’ colonization, and inhibits gene expression of *P. expansum* patulin biosynthetic cluster in colonized apples. (**a**) Michaelis–Menten kinetics analysis tested with 0.3 μM of bacterial lactonase (PPH-G55V) and Patulin at pH 7.5, 25 °C. (**b**–**d**) The addition of 2 μM PPH-G55V to *P. expansum* spores resulted in reduced colonized area. (**b**) Non-infected apples, (**c**) apples infected with *P. expansum* cultures, (**d**) apples infected with *P. expansum* after cultures incubation with 2 μM PPH-G55V. Pictures were taken three days post-infection. (**e**) Lesion size in cm2 of treated apples after 3 days inoculation with *P. expansum*. Mean values are presented (*** *p* < 0.0005, ** *p* < 0.0047 according to one-way ANOVA followed by Tukey–Kramer). (**f**) The relative expression levels of patulin biosynthesis pathway genes in infected apples following PPH-G55V enzymatic treatment (in black) were normalized to the housekeeping gene 28 s at the leading edge of the decay observed after 6 days of inoculation. Data points represent the means of three biological replicates ± standard error. Statistical analysis according to one-way ANOVA (*p* value < 0.05 *, <0.003 **). Fungi treated with the enzyme activity buffer was used as control in gray. ns- Indicates not significant.

Following the UV absorbance of lactone-based mycotoxin patulin from *P. expansum*, at 278 nm, with extension coefficient of 8000 OD/M, (see Figure S1), enabled the detection of enzymatic activity of recombinant expressed and purified PPH-G55V with patulin (Figure 1a). The bacterial enzyme exhibited considerably high catalytic efficiency, with a *k_cat_* value of 0.724 ± 0.077 s^−1^ and K_M_ value of 116 ± 33.98 μM. The calculated specific activity (*k_cat_*/K_M_) showed a value of 6.24 × 10^3^ s^−1^M^−1^, which is one order of magnitude lower than its activity with bacterial QS molecules AHLs [26].

Colonization pattern of *P. expansum* spores mixed with purified PPH-G55V before inoculation induced a 65% (*** *p* < 0.0005) reduction of the lesion area in infected apples after three days (Figure 1b–d). Pre-inoculation spray of 2µM PPH-G55V reduced the lesion area by 46% (** *p* < 0.0047), while post inoculation treatment of the fruit with 2µM PPH-G55V spray, showed no effect on lesion development (Figure 1e).

Analysis of the effect of the fungal–enzyme mix before inoculation on the gene expression in the biosynthetic cluster of patulin during fungal colonization showed a relative inhibition ranging between 28% to 82% (Figure 1f). Relative expression of PatH (encoding m-Cresol methyl hydroxylase), PatI (encoding m-Hydroxybenzyl alcohol hydroxylase), PatF (encoding neopatulin synthase), PatO (encoding Putative isoamyl alcohol oxidase), and PatE (encoding Glucose-methanol-choline), were significantly downregulated by the following percentages compared with the control without enzyme: 78%, 71%, 28%, 82%, and 58%, respectively (*p* < 0.05 *, <0.003 **). 

### 3.2. Bacterial Lactonase Modulate Fungal Growth of P. expansum and Gene Expression in Culture

**Figure 2:**
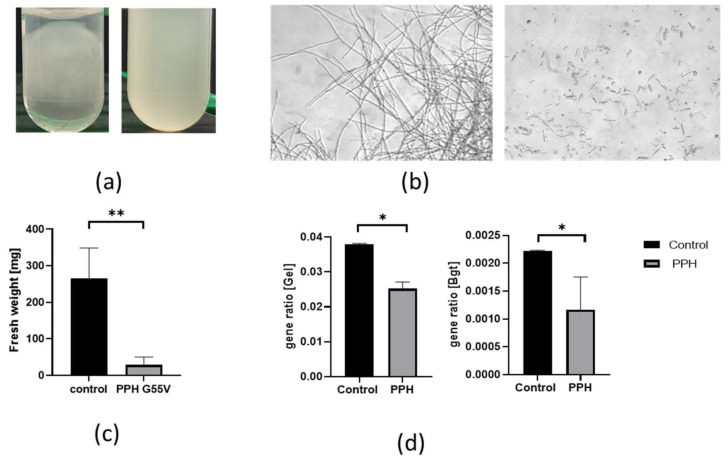
Purified stabilized bacterial lactonase (PPH-G55V) reduced mycelium production and modulated its morphology in PDB medium. (**a**) The addition of 2 μM PPH-G55V bacterial lactonase to a PDB medium containing ~2500 spores of *P. expansum*, reduced mycelium production after three days (right tube), compared with untreated culture (left tube). (**b**) Microscopic picture (×10), presenting the differences in fungal mycelia development between untreated mycelia (left) and PPH-G55V-treated mycelia (right). (**c**) Fungal mycelium fresh weight was significantly lower in the presence of the lactonase than untreated fungi. (** *p* =0.0090, t test) (**d**) Expression levels of genes (Gel and Bgt) normalized to housekeeping gene 28 s. Data points represent the means of three biological replicates ± SE. Statistical significance according to one-way ANOVA comparison (*p* value = 0.0267 * left, *p* = 0.0435 * right). Fungi treated with enzyme activity buffer used as control.

The addition of purified PPH-G55V to *P. expansum* spores grown on PDA solid media did not show any significant change in germination or colony development, see Figure S2. Growth of *P. expansum* spores performed in PDB liquid medium in the presence of the purified enzyme PPH-G55V showed a different pattern of hyphal morphology after three days of growth (Figure 2a, right tube). Microscopic observations indicated thinner cell walls in hyphae grown with PPH-G55V than the hyphae from the fungal culture without the enzyme (Figure 2b). The fresh weight of the fungal mycelium grown with the enzyme showed a ten-fold reduction compared with untreated mycelia (Figure 2c). Sampling the treated mycelia and plating on fresh PDA plates showed apparent differences in the number of new colonies developed from the enzyme-treated mycelia. While hundreds of new small colonies developed from enzyme-treated mycelia, only about 30 colonies developed from control mycelial suspension (Figure S3), suggesting an effect of the enzyme on mycelia.

Next, we tested the expression of several previously shown genes involved in fungal cell wall biosynthesis and morphogenesis. Such genes were identified in *P. chrysogenum* and *P. expansum*. In *P. chrysogenum* the genes are Bgt1 (Pc15g01030), accession number XP_014536515.1 and Gel1 (Pc13g08730), accession number XP_014538414.1, encoding for β(1-3) glucanosyltransferases [63]. These enzymes play a role in the biosynthesis of fungal cell wall by elongating and remodeling β-1,3 glucan. In a recent predicted secretome analysis of *P. expansum* in fruit apple interactions, PEX2_048400, coding for β(1-3)-transglycosylase was highly expressed [64]. Therefore, based on XP_014536515.1 and XP_014538414.1 amino acid sequences, homologs were identified in the *P. expansum* genome (XP_016597554.1 and XP_016594412.1 with 89% and 94%, respectively), using NCBI protein blast. Primers were designed (see primers sequence in Table S1). Next, qRT-PCR analysis indicated that both genes encoding homologs to Gel1 and Bgt1 were downregulated significantly by 37% and 48%, respectively, in enzyme-treated mycelia (* *p* = 0.0267, * *p* = 0.0435; see Figure 2d), suggesting a possible effect of the enzyme on the cell wall biosynthesis of the *P. expansum* hyphae.

### 3.3. Identification of a Putative Lactonases in Fungal Species and Verification of Activity with Patulin for the Homolog from P. expansum

**Figure 3:**
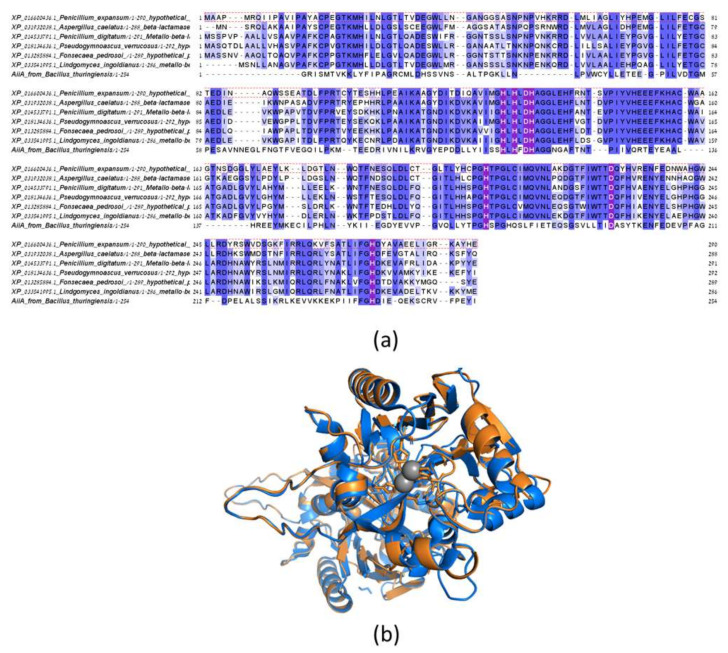
The identification of putative lactonases in various fungal species based on sequence homology and structural modeling of *P. expansum homolog*. (**a**) Multiple-sequence alignment of newly identified putative fungal lactonases. The color intensity correlates with the percentage identity. The HxHxDH~H~D~H motif is common to all AHL lactonases in the MBL (Metallo-β-lactamase) superfamily. The first sequence is of the homolog from *P. expansum*. The residues that coordinate the two catalytic metals are highlighted in dark purple. (**b**) Shown are the backbones of the structural homology models of the putative lactonase from *P. expansum,* PELa (orange) and AHL lactonase from *Alicyclobacillus acidoterrestris* (pdb 36cgy) (blue), and the active-site zinc atoms (gray). The catalytic residues of both enzymes are presented as sticks in the binuclear catalytic center.

**Figure 4:**
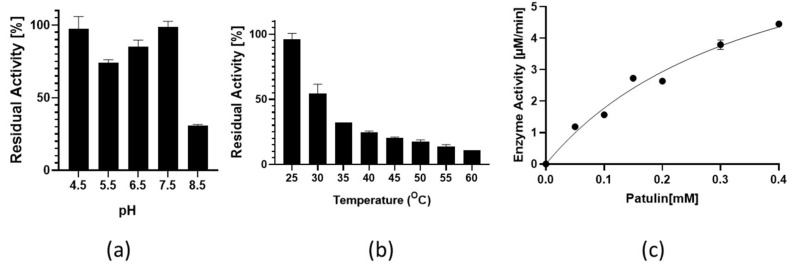
Biochemical characterization of *P. expansum* newly identified enzyme (PELa). The activity of recombinantly expressed and purified PELa from *P. expansum* was tested at different pH levels (**a**) and different temperatures (**b**). Error bars indicate standard deviation of three replicates for each treatment. (**c**) Michaelis–Menten kinetics analysis with 0.6 μM of fungal lactonase in activity buffer, 100 mM Tris-HCl pH 7.5, 100 mM M NaCl, and 100 µM ZnCl_2,_ and 0–0.4 mM patulin in activity buffer pH 7.5, at 25 °C.

As mentioned, one of the hypotheses regarding the role of bacterial AHL lactonases is that they self-regulate QS signaling within the same species, supported by the evidence that AHL-producing bacterial strains can also degrade them [38]. We hypothesized that similarly, fungal-secreting patulin might encodes for a lactonase that degrade patulin for self-regulation or mycotoxin recycling. BLAST analysis using the amino acid sequence of bacterial AHL lactonase, PPH, as the query sequence; did not yield any homologs with above 28% identity in the NCBI-available genomes of *P. expansum*. However, a homolog was identified in *P. expansum* 072460, when the sequence of AiiA from *Bacillus thuringiensis* (WP_000216581.1) belonging to the Metallo-β-Lactamase (MBL) superfamily [65] was used as the query sequence. The homolog shared 29% identity and 76% coverage with an E value of 9 × 10^−17^. Similarly, homologs were identified in other 32 fungal species sharing 60–80% identity between them, see Figure S4. Figure 3a presents the sequence alignment of AiiA from *Bacillus thuringiensis* and fungal species such as *P. expansum*, *Aspergillus clavatus*, *Penicillium digitatum*, *Pseudogymnoascus verrucosus*, *Fonsecaea pedrosoi*, and *Lindgomyces ingoldianu*. The alignment indicates that the newly identified fungal proteins are putative lactonases as they all possess a signature sequence, the HxHxDH~H~D~H motif, common to lactonases in the MBL superfamily [65]. A 3D homology model was predicted based on the amino acid sequences of the homolog from *P. expansum* using the solved structure of an AHL lactonase from *Alicyclobacillus acidoterrestris* (PDB 36cgy) as template. As shown in Figure 3b, the structural overlay of the metal ion-coordinating residues in the active site of AHL lactonase from *Alicyclobacillus acidoterrestris* and the homology model of *P. expansum*, indicates that the two proteins share the same fold and bear a similar active site. Therefore, the synthetic gene of the homolog from *P. expansum* dubbed here PELa was cloned into an expression vector, recombinant expressed, and purified. The newly identified enzyme maintained its activity between pH values of 4.5–7.4, and its highest activity was detected at 25 °C (Figure 4a,b). Michaelis–Menten analysis of the activity with patulin (Figure 4c) showed a *kcat* value of 0.235 ± 0.002 s^−1^, K_M_ value of 376.7 ± 112 μM, and the calculated specific activity *k_cat_*/K_M_ value of 6.24 × 10^2^ s^−1^M^−1^, an order of magnitude lower activity than that observed with PPH-G55V. These results indicate that *P. expansum* lactonase might be involved in self-regulation by patulin recycling. Towards the understanding of the involvement of lactonase activity with an ecological relevant bacteria in degrading fungal mycotoxins in the apple microbiome, the sequence of AiiA from *Bacillus thuringiensis* (WP_000216581.1) was used similarly to identify putative lactonases from bacteria that reside in apple trees, such as in *Bacillus megaterium* (basionym: *Priestia megaterium*). *B. megaterium* was identified in bark samples from apple and pear orchards and was suggested to be antagonistic to the AHL-producing pathogen *Erwinia amylovora E. amylovora*) [66]. Indeed, a homolog sharing 96% identity was identified, with the accession number ACX55098.1 annotated as AHL lactonase by NCBI.

## 4. Discussion

The apple microbiome is comprehensively studied, and recent studies have shown that different apple fruit tissues (calyx-end, stem-end, peel, and mesocarp) harbor distinctly different fungal and bacterial communities that vary in diversity and abundance [1,47,51,67,68]. However, few studies have focused on understanding the molecular mechanisms involving the interactions between epiphytic microbial (both bacterial and fungal) populations [1]. One of the questions is related to the understanding of specific interactions, including metabolites and enzymes. This can increase the knowledge of using microbial antagonists as an alternative to synthetic chemicals in managing apples’ postharvest bacterial and fungal pathogens.

Recently, an efficient AHL lactonase was identified and characterized in phytopathogen *Erwinia amylovora*[56]. Furthermore, adding this purified enzyme to *Erwinia amylovora* cultures resulted in a lower relative expression level of bacterial QS-regulated genes [56]. However, the ability of such bacterial lactonase to degrade lactone-based fungal mycotoxins was not explored, nor was the effect on fungal cultures, gene expression, and virulence. Here, we used a highly active and stable AHL lactonase (PPH-G55V) [53] to test these effects. Our results suggest a new function for bacterial AHL lactonases, with a hydrolytic mechanism, thus far known to degrade mainly bacterial AHLs, QS signaling molecules and act as quorum-quenching enzymes that reduce virulence plant pathogens [27,32,33,69,70]. Patulin is a lactone-based fungal mycotoxin, shown to be related to pathogenicity affecting mycelium growth, and linked to host-pathogen-microbe interaction [58,71,72,73]. We tested patulin degradation with the bacterial AHL lactonase. To test the bacterial enzyme effect on fungi culture and during apple infection, we recombinant expressed and purified the stabilized PPH-G55V. Present findings indicate that PPH-G55V could degrade patulin with a *k_cat_*/K_M_ value of 6.21 × 10^3^ s^−1^M^−1^, one to two orders of magnitude lower activity than its high efficiency with the bacterial AHLs [26]. At the same time, the capability of the bacterial lactonase to reduce pathogenicity *in planta* confirmed patulin suggested role as a factor contributing to pathogenicity of *P. expansum*, and the ability of this lactonase to reduce infection in apples.

Furthermore, the bacterial AHL lactonase added to fungal cultures inhibited the relative expression level of genes involved in patulin biosynthetic cluster during apple tissue colonization by *P. expansum*. The inhibited genes included PatH (m-Cresol methyl hydroxylase), PatI (m-Hydroxybenzyl alcohol hydroxylase), PatO (Putative isoamyl alcohol oxidase), and PatE (Glucose-methanol-choline). The inhibition ranged from 28 up to 82%. Interestingly, the last precursors in patulin synthesis such as neopatulin and ascladiol possess lactone rings [9], and the gene expression of their corresponding enzymes was significantly inhibited. This indicates that the bacterial lactonase may not only degrade patulin but also affect its biosynthetic pathways at the gene expression level, in a mechanism that is yet to be discovered. Recently, the bacterial *Methylobacterium oryzae* co-cultured with *P. expansum*, showed an inhibition of *P. expansum*, on patulin production and on the transcriptional level of the gene coding for Isoepoxydon dehydrogenase [24].

**Scheme 1:**
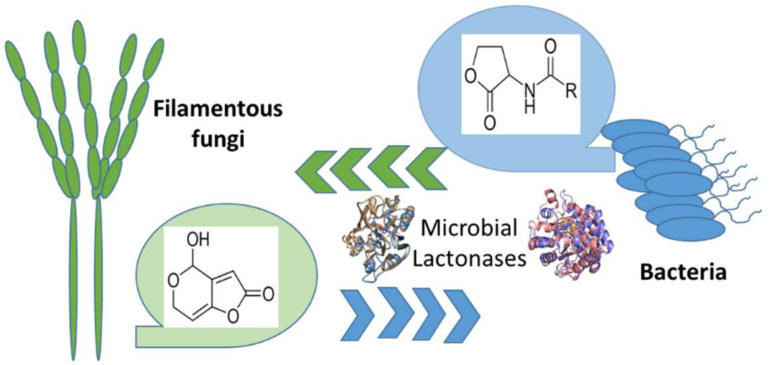
Lactone-based patulin and AHLs in fungal and bacterial species. The ability of lactonase to degrade these lactones affect gene expression and virulence in both bacteria and fungi; suggest patulin degradation by lactonases has an ecological role in both fungal and bacterial species.

The bacterial enzyme PPH-G55V also showed a significant effect on the morphology of *P. expansum* growth. While the addition of bacterial enzyme PPH-G55V did not inhibit fungal growth in solid media, and no effect seen on germination, it did affect fungal development and mycelia production when added to mycelia in liquid media. It further reduced the expression of Gel1 and Bgt1 homologs, coding for enzymes known to be involved in fungal cell wall development to 66% and 52%, respectively. Interestingly, the regeneration of colonies from enzyme-treated mycelia in liquid culture resulted in a multi-development of colonies compared with the control. These results suggest that the lactonase activity induces a morphological change in liquid growth related to cell wall development. Therefore, we surmise a link between the reduction in *P. expansum* apple colonization caused by the addition of bacterial lactonase to fungal cultures, patulin metabolism and the changes in morphology via the downregulation of the biosynthetic cluster of patulin and cell wall development-related genes. These results also suggest that AHL lactonases may interfere with inter-kingdom communication between fungal and bacterial communities via their ability to degrade lactone-based mycotoxins (Scheme 1). As there are growing numbers of studies indicating that fungal mycotoxins play an essential role in inhibiting bacterial communications such as QS [46], the presented results suggest that bacterial AHL lactonase may disturb fungal communication signals in the apple microbial environment, acting in an antagonistic mechanism. Although *M. tuberculosis* (PPH bacterial origin) is not an epiphytic apple bacterium, we have previously identified and characterized a lactonase in an apple bacterial pathogen, *E. amylovora*, as a quorum-quenching lactonase [56]. This bacterium is part of the epiphytic community in apples and pears trees; furthermore we identified herein a putative lactonase sequence in *Bacillus megaterium*. Future work should test the effect of lactonases naturally expressed in bacteria co-cultured with *P. expansum*.

Since some bacteria that secret AHLs also express AHL lactonases, we surmised that the fungi would have patulin degrading lactonase activity. Indeed, putative lactonases homologs from the MBL (metallo-β-lactamase) fold were identified in various fungal species, and the activity of the recombinant expressed and purified homolog from *P. expansum* was verified with patulin, but with an order of magnitude lower activity than the activity measured with the bacterial lactonase. 

While postharvest biocontrol products using microbial antagonists, especially yeasts, have been isolated from epiphytic communities of the fruits and commercialized, wide use is limited due to problems with efficacy and regulatory hurdles [74]. A greater understanding of the fruit microbiome is needed to elucidate the factors involved in biocontrol systems. This would facilitate improved strategies that rely on antagonistic microorganisms or enzymes for managing postharvest diseases of fruit crops [1,48,75]. The results presented here suggest that bacterial lactonases may be one mechanism used by antagonist species, and purified and stable enzymes may serve as a better strategy than using antagonistic bacteria, reliving microbial competition. Specifically, PPH-G55V can be further developed as pretreatment to reduce fungal damaged apples, by applying the enzyme on apples before storage. Another possible application is as a treatment for high patulin residual concentration in apple products.

## 5. Conclusions

Bacterial QQ lactonases, thought to evolve towards the degradation of bacterial AHL molecules, are widely conserved in various bacterial species and have a variable substrate range. Here, the ability to degrade patulin by one such bacterial enzyme from the PLL family was described. Patulin is a lactone-based fungal mycotoxin. This lactonase activity appears to be correlated with inhibiting fungal colonization due to interfering with patulin concentration and synthesis, and cell wall morphology. The lactonase inhibitory effect is supported by reducing relative gene expression upon its addition to *P. expansum* cultures. Understanding the impact of patulin on beneficial or harmful microorganisms that reside within the microbiome and its enzymatic degradation can identify new antimicrobial methods to reduce fruit decay and decrease mycotoxin contamination.

Moreover, the degradation of patulin by bacterial lactonase presents a new method to study the interaction between bacteria and fungi communities. Patulin hydrolyzing activity by epiphytic bacteria can be referred to as part of inter-kingdom communication between fungi and bacteria. On the other hand, fungal lactonases might play a role in fungal self-regulation of patulin synthesis. Present results also suggest a potential application of QQ lactonases with patulin-degrading activity as a new approach for disease control of postharvest infection by *P. expansum* and other postharvest pathogens producing lactone mycotoxins. 

## Data Availability

Data is contained within the article or supplementary material.

## Supplementary Materials

The following are available online at https://www.mdpi.com/article/10.3390/jof7100826/s1, Figure S1: Patulin absorbance scan and a calibration curve at 278nm to enable enzymatic activity detection, Figure S2: The addition of purified PPH-G55V (bacterial lactonase) to P. expansum spores did not show any significant change in germination or colony development. Figure S3: Purified bacterial lactonase (PPH-G55V) effect fungal morphology after extraction of the hypha from liquid medium. Figure S4: Multiple-sequence alignment of newly identified putative fungal lactonases, Table S1: Oligonucleotides used for qPCR study.
