# Supplementary material for: Bacterial Quorum-Quenching Lactonase Hydrolyzes Fungal Mycotoxin and Reduces Pathogenicity of Penicillium expansum—Suggesting a Mechanism of Bacterial Antagonism
Source: J Fungi (Basel). 2021 Oct 2;7(10):826. doi: 10.3390/jof7100826 (PMC8537011; doi:10.3390/jof7100826)
Supplement: Supplementary file 1 [file jof-07-00826-s001.zip › jof-1390238-supplementary.pdf]

Journal of *Fungi*

MDPI

Supplementary Material of:

Bacterial Quorum-Quenching Lactonase Hydrolyzes Fungal Mycotoxin and Reduces Pathogenicity of *Penicillium expansum*—Suggesting a Mechanism of Bacterial Antagonism

Shlomit Dor [1], Dov Prusky [2] and Livnat Afriat-Jurnou [1,3,*]

[1] Migal-Galilee Research Institute, Kiryat Shmona, 11016, Israel; shlomitd@migal.org.il

[2] Department of Postharvest Science, Agricultural Research Organization, Rishon LeZion, 7505101, Israel

[3] Faculty of Sciences and Technology, Tel-Hai Academic College, Upper Galilee, 1220800, Israel

* Correspondence: dovprusk@volcani.agri.gov.il dovprusk@agri.gov.il (D.P.); livnatj@migal.org.il (L.A.-J.)

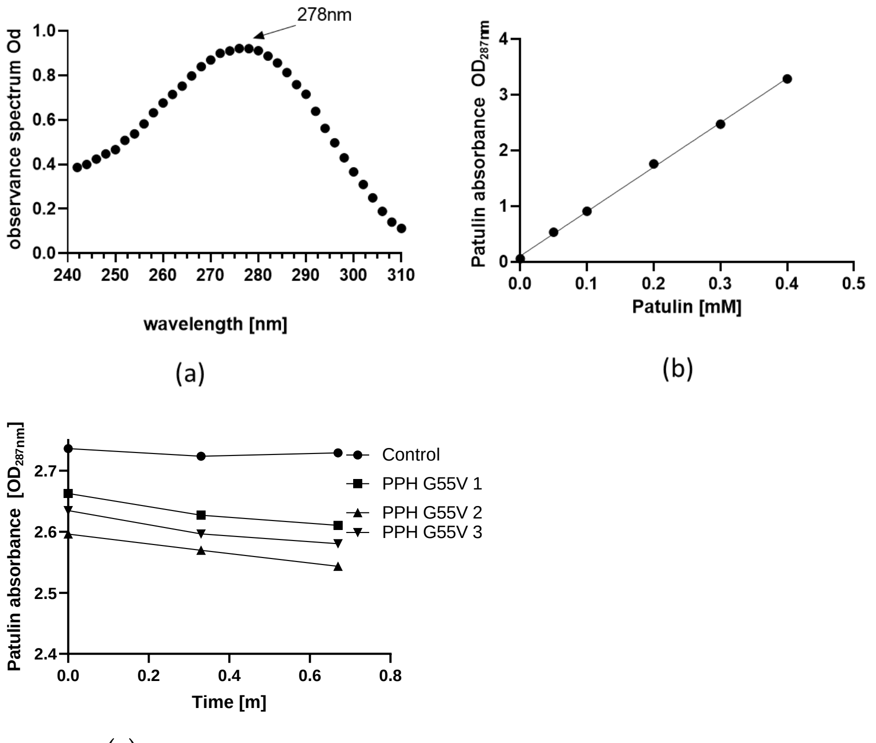

(c)

Figure S1: **Patulin absorbance scan and a calibration curve at 278nm to enable enzymatic activity detection.** (**a**) Absorbance spectrum scan (240-310 nm) of a solution containing 0.1mM patulin dissolved in enzyme activity buffer containing 100mM Tris pH 7.5, 100mM NaCl, 100uM $MnCl_2$. (**b**) The absorbance tested at 278nm of patulin solution at concentration ranging from 0-0.4mM (in 100mM Tris pH 7.5, 100mM NaCl, 100uM $MnCl_2$.) was used to calculate the extension coefficient from the linear fit Y = 7.983*X + 0.1041 R2 = 0.9988. (**c**) Enzymatic activity analysis tested with 0. 3 μM of PPH-G55Vand 0.25mM patulin in activity buffer (100mM Tris pH 7.5, 100mM NaCl, 100uM $MnCl_2$), at 25 °C.

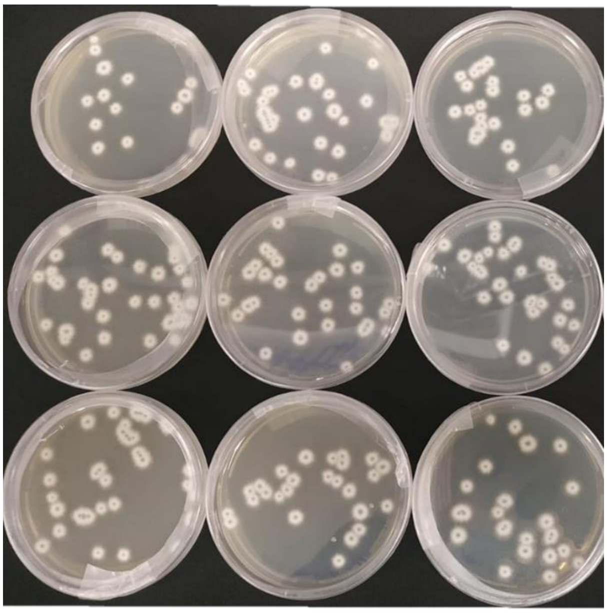

(a) (b) (c)

Figure S2: The addition of purified PPH-G55V (bacterial lactonase) to *P. expansum* spores did not show any significant change in germination or colony development. Spores were grown on PDA solid media. (**a**) *P. expansum* spores alone (**b**) Spores incubated with 1μM PPH-G55V (**c**) Spores incubated with enzyme activity buffer (100mM Tris-HCl pH 7.5, 100mM NaCl, 100μM MμCl$_2$).

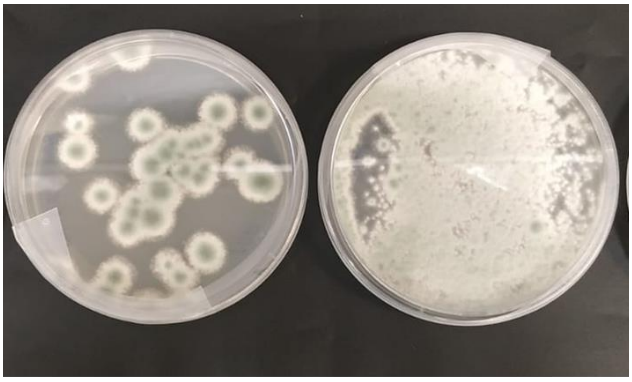

(a) (b)

Figure S3: Purified bacterial lactonase (PPH_G55V) effect fungal morphology after extraction of the hypha from liquid medium. (**a**) Hypha of *P. expansum* culture alone (b) Hypha *P. expansum* culture treated with purified PPH-G55V. Presenting 1 representative picture of 3 repeats. Pictures were taken after 5 days incubation.

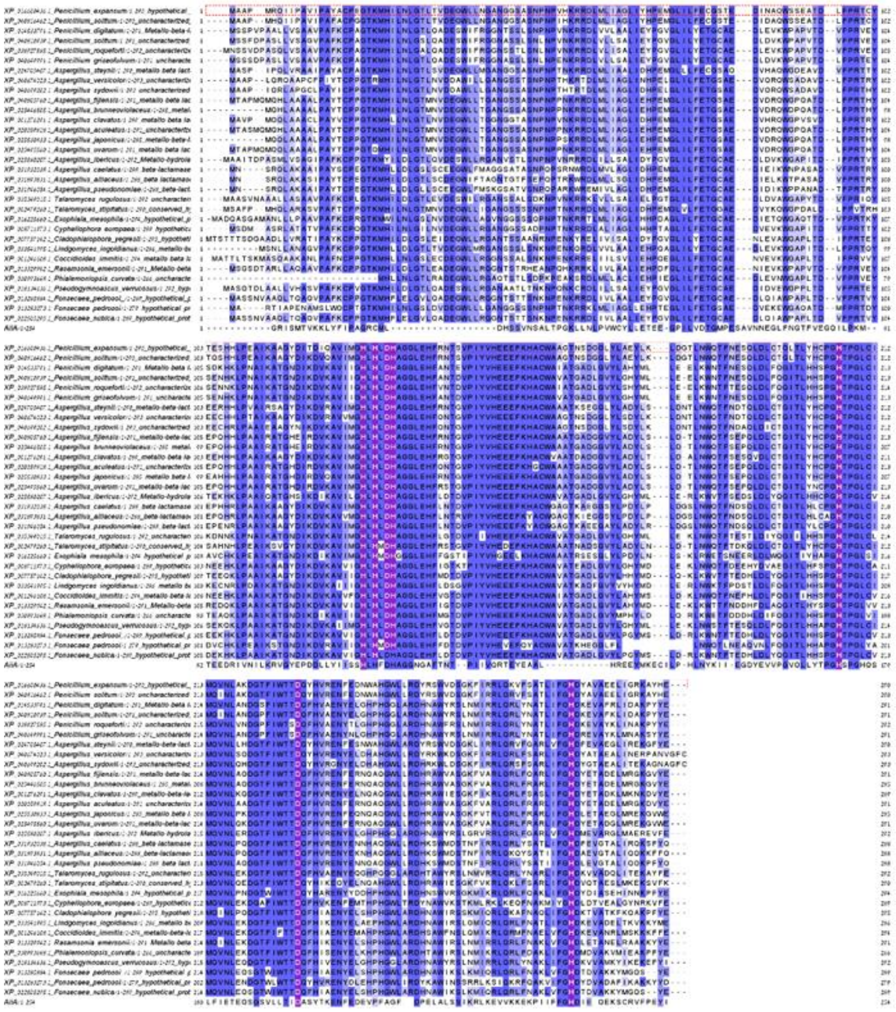

Figure S4: Multiple-sequence alignment of newly identified putative fungal lactonases. The color intensity correlates with the percentage identity. The HxHxDH~H~D~H motif is common to all AHL lactonases in the MBL superfamily. The residues that coordinate the two catalytic metals are highlighted in dark purple.

Supplementary Table S1. Oligonucleotides used for qPCR study

Oligonucleotides	Nucleotide sequences (5′-3′)	
F28S	GGAACGGGACGTCATAGAGG	Brad et al 2016 [1]
R28S	AGAGCTGCATTCCCAAACAAC	Brad et al 2016
PatN_F-RT	CGTTCGATGTCGCTAGCAAA	Brad et al 2016
PatN_R-RT	GGCGATAATCACGTCAATTCG	Brad et al 2016
PatF_F-RT	ATGAAATCCTCCCTGTGGGTTAGT	Brad et al 2016
PatF_R-RT	GAAGGATAATTTCCGGGGTAGTCATT	Brad et al 2016
PatE_F-RT	CATTCTCATCGGGCCTGAGT	Brad et al 2016
PatE_R-RT	TCGAAGCTCTTCCGGACATG	Brad et al 2016
PatI_F-RT	GCAAACTCATTCCGCAAGGA	Brad et al 2016
PatI_R-RT	TGGTTCTTGCCATCGATCAC	Brad et al 2016
PatH_F-RT	CATTTATCGGCGGTGTTCTGA	Brad et al 2016
PatH_R-RT	GATCAACGCTTGCACGATAGC	Brad et al 2016
PatO_F-RT	TCGCCTCCTGGTGTGTATCTT	Brad et al 2016

PatO_R-RT	AAGCGTGCCCAGTCATTCAG	Brad et al 2016
PatK_F-RT	GACGCTGGGCTACTGGATTG	Brad et al 2016
PatK_R-RT	TCGTGCGTGAGGCCAGTAT	Brad et al 2016
PatL_F-RT	GCAGGAGATCCGTTTCAGACA	Brad et al 2016
PatL_R-RT	CCACTGACCGACGGTTACAAC	Brad et al 2016
Gel1 F	ATGACCGTTTCTACATGCGC	current work
Gel1 R	CATCGTGGTCCTGGGAGTTA	current work
Bgt1 f	CCACTCTCGTCCGTACCTAC	current work
Bgt1r	TTCTTGGTGTCGGTAGGCTT	current work

1. Barad, S.; Espeso, E.A.; Sherman, A.; Prusky, D. Ammonia activates pacC and patulin accumulation in an acidic environment during apple colonization by Penicillium expansum. *Mol. Plant Pathol.* **2016**, doi:10.1111/mpp.12327.
